# Effectiveness and safety of surgical interventions for treating adolescent idiopathic scoliosis: a Bayesian meta-analysis

**DOI:** 10.1186/s12891-020-03233-1

**Published:** 2020-07-02

**Authors:** Long Chen, Zeyu Sun, Jingming He, Yunwen Xu, Zhuhai Li, Qian Zou, Bo Li

**Affiliations:** 1 Department of Orthopedics, Guizhou Provincial People’s Hospital, No.83 Zhongshan East Road, Guiyang, 550000 Guizhou China; 2 Department of Epidemiology, Johns Hopkins Bloomberg School of Public Health, Baltimore, MD 21205 USA; 3 Department of Orthopedics, The People’s Hospital of Guangxi Zhuang Autonomous Region, Nanning, 530021 Guangxi China

**Keywords:** Adolescent idiopathic scoliosis, Surgical interventions, Pulmonary function, Complications, Cobb angle, Bayesian meta-analysis

## Abstract

**Background:**

Adolescent idiopathic scoliosis (AIS) is the most common form of spinal deformity in children and adolescents which presents as complex three-dimensional (3D) deformity of the spine and rib cage. This study aimed to estimate the effectiveness and safety of surgical interventions for AIS using Bayesian meta-analysis.

**Methods:**

The PubMed, EMBASE, and Cochrane Controlled Register of Trials were searched through Oct 1, 2019, without language restrictions. Relevant studies evaluating combined effectiveness and safety of surgical interventions for AIS were included according to eligibility criteria. The primary outcome measures included pulmonary function (change of absolute forced vital capacity and forced expiratory volume in 1 second from pre-operation to post-operation) and incidence of complications. The secondary outcome measure was change of Cobb angle from pre-operation to post-operation. Data was pooled using a random effects model in pairwise meta-analysis. Bayesian meta-analysis combined direct and indirect evidence using a Bayesian framework.

**Results:**

Twenty-eight case-controlled studies with totally 1970 participants were included. This Bayesian meta-analysis combining direct and indirect evidences indicated that posterior fusion with instrumentation without thoracoplasty (PSF) had the highest probability to achieve better pulmonary function and lower complication rate; video assisted anterior fusion with instrumentation without thoracoplasty (VAT) had the highest probability to obtain better Cobb angle correction based on analysis of rank probability.

**Conclusion:**

This Bayesian meta-analysis demonstrated that PSF had the highest probability to achieve better post-surgical pulmonary function and lower complication rate, which gives a practical recommendation of PSF as a primary surgical treatment for AIS. The results also support statistics that current surgeries adopted more PSF but less open anterior approach surgery and thoracoplasty. More research work is required to address the effectiveness and safety of VAT for treating AIS more convincingly.

## Background

Adolescent idiopathic scoliosis (AIS) is the most common form of spinal deformity in children and adolescents which presents as complex three-dimensional (3D) deformity of the spine and rib cage [1–3]. Its prevalence is about 1–3% of adolescent aged 10 to 16 years old, with almost 10% and up to 0.1% of the patients having the necessity of treatment and surgery, respectively [4]. The diagnosis of AIS is traditionally based on Cobb method for evaluation of the spinal curvature. Patients are diagnosed as AIS when the Cobb angle ≥10° [5]. Treatment strategies for AIS can be conservative or surgical. Conservative treatments including observation and brace treatment are always applied to patients with small and moderate curves or skeletal maturity, otherwise, surgical treatment would be recommended for those with severe curves [6].

Surgical interventions aim to terminate the progression of AIS, achieve maximum permanent correction of the 3D deformity, promote appearance by balancing the trunk, and keep lower incidence rate of short-term and long-term complications [7]. The surgery for AIS has been developed for one century since Hibbs first performed fusion procedure to treat AIS [8]. In 1953, Harrington introduced instrumentations to spinal fusions, which could improve the correction of deformity and decrease the pseudarthrosis rate [9]. The postoperative cast had been a routine procedure in use until Luque’s sublaminar wiring was introduced to treat AIS in 1970s [10]. Although several methods were introduced, thoracotomy was still required before 2000 because of the rib prominence and coronal plane decompensation [4]. Then segmental hook instrumentation, segmental pedicle screw constructs and hybrid constructs (pedicle screws, hooks, and wires) were successively introduced to three dimensional correction for treating AIS [11]. Depending on specific conditions diagnosed in clinic, different surgical procedures are adopted. The posterior surgery technique was developed by Harrington in 1962, which have been improving with clinical practice till recently and become a widely used standard procedure for treating AIS [12, 13]. Anterior surgery was reported by Dwyer et al. in 1974, which has been used commonly for thoracolumbar and lumbar curves [14]. In order to reduce the adverse effects for pulmonary function after open thoracotomy and anterior spinal fusion, thoracoscopic and video assisted procedures were developed for treating AIS [15]. As recently summarized by Lee et al. [16], the common surgical interventions for treating AIS were categorized as follows: combined anterior release and posterior fusion with instrumentation (ASR + PSF), combined video assisted anterior release and posterior fusion with instrumentation without thoracoplasty (VAT+PSF), posterior fusion with instrumentation without thoracoplasty (PSF), anterior fusion with instrumentation and thoracotomy without thoracoplasty (ASF), video assisted anterior fusion with instrumentation without thoracoplasty (VAT), and any scoliosis surgery with additional thoracoplasty or multiple convex rib resections (WT). To choose the safest and the most effective procedures has always been essential for patient treatments.

Several traditional pairwise meta-analyses of surgical interventions for treating AIS have been published previously [2, 13, 16, 17]. The information provided by these studies is limited because the traditional pairwise meta-analysis is only capable of making comparison between two surgical interventions at a time, thus prone to result in a local optimum as the conclusion. To compare effectiveness and safety across multiple surgical interventions at a time, taking the advantages and disadvantages of all the procedures into consideration, would generate more comprehensive criterion for decision-making of the doctors. To achieve this, Bayesian meta-analysis was developed as an attractive evidence-based technique to compare the relative benefits of multiple interventions and obtain rank probability of these interventions [18], which have overcome the limitation of traditional pairwise meta-analysis. In contrary to traditional meta-analysis, in which only direct evidence is available, Bayesian meta-analysis could also incorporate indirect evidences among all interventions besides direct evidences to improve estimation precision.

The objective of this study is to compare the effectiveness and safety of ASR + PSF, VAT+PSF, PSF, ASF, VAT and WT for treating AIS via Bayesian meta-analyses regarding 4 criteria: Cobb angle, absolute forced vital capacity (FVC), absolute forced expiratory volume in 1 second (FEV1) and incidence of complications.

## Methods

This Bayesian meta-analysis was performed according to Preferred Reporting Items for Systematic Reviews and Meta-Analyses (PRISMA) guidelines [19], and was also registered on PROSPERO (CDR 42018079968).

### Data sources and searches

Databases including Cochrane Controlled Register of Trials, PubMed and EMBASE (Jan 1980 to Oct 2019) were used to identify all studies that evaluated the effectiveness and safety of surgical interventions for treating AIS, with the searching strategy being: (Adolescent idiopathic scoliosis) AND (surgery OR surgical intervention OR surgical treatment OR surgical management) AND (randomized controlled trials OR case-controlled trials). The titles and abstracts were screened by two independent reviewers to exclude any reports that did not assess the effectiveness and safety of surgical treatment for AIS. Full texts of the remaining articles were reviewed to identify studies that met inclusion criteria.

### Inclusion and exclusion criteria

The inclusion criteria were: (1) target population: pediatric patients suffered from AIS; (2) interventions: ASR + PSF, VAT+PSF, PSF, ASF, VAT and WT for treating AIS; (3) methodological criteria: Controlled clinical trials.

The exclusion criteria were: (1) target population: patients with other type of scoliosis; (2) interventions: conservative treatments or surgical treatments except those that were above-mentioned; (3) methodological criteria: case series.

### Outcome assessment

The primary outcome measures include pulmonary functions (change of absolute FVC and FEV1 from pre-operation to post-operation) and incidence of complications (including infection, hook and screw dislodgement, hemothorax, pleural effusion, neurological complications, vascular complications, etc.). The secondary outcome measure was change of Cobb angle from pre-operation to post-operation.

### Data extraction and assessment of risk of bias

For each trial, we gathered data on study type, sample size, interventions and follow-up. In addition, the following clinical data were also extracted if available: the Cobb angle from pre-operation and post-operation to calculate the change of Cobb angle, absolute FVC and FEV1 from pre-operation and post-operation to calculate the change of absolute FVC and FEV1, and incidence of complications. Two researchers extracted the data independently according to the pre-specified inclusion and exclusion criteria. Disagreements were resolved by discussion.

The Newcastle-Ottawa Scale was used to assess the quality of case-controlled trials in terms of selection and comparability of the study groups, and determination of outcomes, with a maximum of nine points using the criteria listed in Table S1 in the Supplement [20].

### Data synthesis and statistical analysis

Pairwise meta-analysis was performed with a random effects model using ADDIS software (version 1.16.6, drugis.org). In each study, the odds ratio (OR) was calculated for dichotomous outcomes, and mean differences (MDs) was calculated for continuous outcomes. Both were presented with 95% confidence interval (CI). The pooled estimates of ORs or standardized MDs and 95% CI of four outcomes (change of Cobb angle, change of absolute FVC, change of absolute FEV1 and incidence of complications) were determined. Heterogeneity in each result was assessed by chi-squared and I^2^ statistic.

Bayesian meta-analysis combined direct and indirect evidences within a Bayesian framework. The Bayesian framework was performed by ADDIS statistical software (version 1.16.6). Convergence was assessed using the Brooks-Gelman-Rubin method. This method compares within-chain and between-chain variance to calculate the potential scale reduction factor (PSRF) for which a value close to “1” indicates approximate convergence has been reached [21]. Inconsistency was detected using the calculation of inconsistency factors and node-splitting analysis, for which an inconsistency factor close to ‘0’ and the 95% CI covers 0 mean that there is no evidence of inconsistency [22]. Node-splitting analysis allows comparing the estimated quantiles for the direct and indirect evidences as well as the combined evidences [23]. In addition, a *p* value was shown and inconsistency would be considered statistically significant when *p* value was less than 0.05. A consistency model was employed for analysis with no evidence of significant inconsistency, otherwise, an inconsistency model was employed in the analysis with evidence of significant inconsistency. Bayesian approach within consistency model would allow ranking the six surgical interventions for treating AIS. The rank probability analysis was adopted to compare interventions, with Rank 1 to Rank 6 representing decreasing positive expectations from the interventions, and all interventions sharing a total possibility of 1 within each rank. For example, an intervention would be certain to be better when it obtained a higher proportion of “Rank 1”, on the contrary, an intervention would be certain to be worse when it obtained a higher proportion of “Rank 6”. Sensitivity analyses that switched the statistical model (consistency and inconsistency model) for each result to calculate the variance parameters were performed to determine whether results were reliable.

## Results

### Study selection

**Fig. 1:**
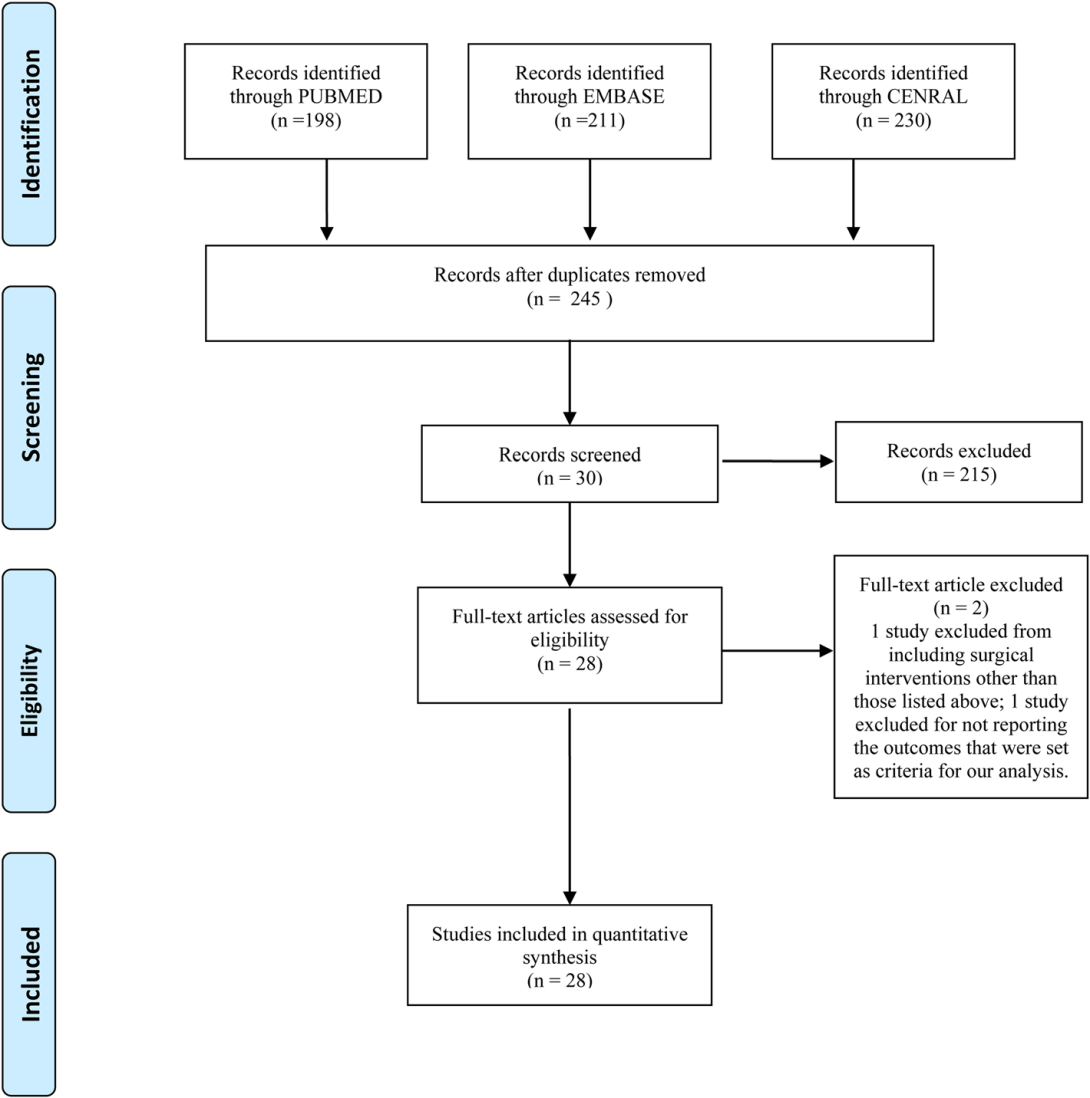
Flow chart Showing Selection of Studies

Figure 1 shows the study selection process. The search strategy retrieved 639 studies in total. The titles and abstracts of these studies were examined by two reviewers, and 30 studies were identified for further analysis. Two studies were excluded, as one [24] included surgical interventions other than those listed above and the other [25] did not report the outcomes that are set as criteria for our analysis. Twenty-eight case-controlled trials [15, 26–52] were considered as relevant studies and were subjected to Bayesian meta-analysis.

### Characteristics and risk of bias of included trials

Table 1 provides a summary of all the 28 studies, with totally 1970 participants included. Among all the participants, 134 patients were assigned to ASR + PSF, 873 to PSF, 399 to ASF, 211 to VAT, 292 to WT and 61 to VAT+PSF. Twenty studies reported change of Cobb angle as an outcome, 11 studies used change of absolute FVC as an outcome, 12 studies reported change of absolute FEV1 as an outcome and 14 studies reported incidence of complications as an outcome.

**Table 1:** Characteristics of the 28 included studies

Source	Study design	Country	Interventions	Sample size	Follow-up (year)	Outcomes for analysis
Dobbs 2006 [26]	Retrospective nonrandomized controlled trial	USA	ASR + PSF vs PSF	20/34	2.4	①
Zhang 2012 [52]	Prospective nonrandomized controlled trial	China	ASR + PSF vs PSF	31/33	2	①, ④
Zhang 2009 [41]	Retrospective nonrandomized controlled trial	China	ASR + PSF vs PSF	32/94	3.4	①
Qiu 2011 [42]	Retrospective nonrandomized controlled trial	China	VAT+PSF vs PSF	18/27	3.65	①, ④
Zhang 2011 [51]	Retrospective nonrandomized controlled trial	China	ASR + PSF vs PSF	12/17	3	①
Kim 2005 [34]	Prospective nonrandomized controlled trial	USA	PSF vs WT vs ASF vs ASR + PSF	49/41/16/12	5	①
Bullmann 2013 [46]	Prospective nonrandomized controlled trial	Germany	ASF vs WT	40/29	2	①, ③, ④
Vedantam 2000 [44]	Prospective nonrandomized controlled trial	USA	PSF vs WT vs ASF vs ASR + PSF	47/33/7/11	2	①, ②, ③
Kim 2008 [33]	Retrospective nonrandomized controlled trial	USA	WT vs ASF	35/29	2	②, ③
Faro 2005 [28]	Prospective nonrandomized controlled trial	USA	VAT vs ASF	31/23	1	①, ②, ③
Kishan 2007 [35]	Prospective nonrandomized controlled trial	USA	VAT vs WT	36/43	2	②, ③
Lenke 2004 [15]	Prospective nonrandomized controlled trial	USA	VAT+PSF vs ASR + PSF	21/16	2	①, ②, ③
Verma 2011 [45]	Retrospective nonrandomized controlled trial	USA	PSF vs ASF vs VAT vs VAT+PSF	70/35/32/22	2	②, ③, ④
Zhou 2011 [49]	Retrospective nonrandomized controlled trial	China	PSF vs WT	24/20	2	①, ②, ③, ④
Greggi 2010 [31]	Retrospective nonrandomized controlled trial	Italy	WT vs PSF	40/40	5	①, ②, ③, ④
Lonner 2009 [37]	Retrospective nonrandomized controlled trial	USA	VAT vs PSF	17/17	2	④
Newton 2013 [40]	Prospective nonrandomized controlled trial	USA	VAT vs ASF vs PSF	55/17/77	2	②, ③, ④
Suk 2008 [43]	Retrospective nonrandomized controlled trial	Korea	PSF vs WT	37/20	2	①, ②, ③, ④
Graham 2000 [30]	Prospective nonrandomized controlled trial	USA	WT vs ASF	31/20	2	②, ③
Miljenko 2006 [29]	Prospective nonrandomized controlled trial	Croatia	ASF vs PSF	25/25	2	①
Lenke 1999 [36]	Prospective nonrandomized controlled trial	USA	ASF vs PSF	70/53	2	①
Wong 2004 [48]	Retrospective nonrandomized controlled trial	Singapore	PSF vs VAT	19/12	2	④
Muschik 2006 [39]	Retrospective nonrandomized controlled trial	Germany	ASF vs PSF	37/104	2	①, ④
Lonner 2006 [38]	Retrospective nonrandomized controlled trial	USA	VAT vs PSF	28/23	2	④
Hee 2007 [32]	Retrospective nonrandomized controlled trial	Singapore	ASF vs PSF	25/11	3.7	①
Wang 2008 [47]	Prospective nonrandomized controlled trial	China	ASF vs PSF	16/16	2	①
Dong 2015 [27]	Retrospective nonrandomized controlled trial	China	ASF vs PSF	17/36	2	①, ④
Zhan 2010 [50]	Retrospective nonrandomized controlled trial	China	ASF vs PSF	22/20	2	①, ④

*AIS* adolescent idiopathic scoliosis; ①: change of Cobb angle; ②: change of absolute forced vital capacity (FVC); ③: change of absolute forced expiratory volume in 1 s (FEV1); ④: incidence of complications; *ASR + PSF* combined anterior release and posterior fusion with instrumentation, *VAT+PSF* combined video assisted anterior release and posterior fusion with instrumentation without thoracoplasty, *PSF* posterior fusion with instrumentation without thoracoplasty, *ASF* anterior fusion with instrumentation and thoracotomy without thoracoplasty, *VAT* video assisted anterior fusion with instrumentation without thoracoplasty, *WT* any scoliosis surgery with additional thoracoplasty or multiple convex rib resections

As assessed by the Newcastle-Ottawa Scale, one case-controlled study [48] was awarded a score of eight points, 18 studies received a score of seven points [15, 26–29, 31–33, 35, 37, 38, 41, 42, 45, 47, 50–52], and nine studies [30, 34, 36, 39, 40, 43, 44, 46, 49] got a score of six points (Table 2).

**Table 2:** Quality assessment of case-controlled studies comparing surgical interventions for treating AIS using Newcastle-Ottawa Scale

Author group		Selection			Comparability		Exposure	
	Adequat-e case definitio-n	Representativeness of the cases	Selectio-n of Control-s	Definitio-n of Controls	Comparability of cases and controls	Ascertainm-ent of exposure	Same method of ascertainmen-t	Non Response rate
Dobbs 2006 [26]	1	1	–	1	2	1	1	–
Zhang 2012 [52]	1	1	–	1	2	1	1	–
Zhang 2009 [41]	1	1	–	1	2	1	1	–
Qiu 2011 [42]	1	1	–	1	2	1	1	–
Zhang 2011 [51]	1	1	–	1	2	1	1	–
Kim 2005 [34]	1	1	–	1	1	1	1	–
Bullmann 2013 [46]	1	1	–	1	1	1	1	–
Vedantam 2000 [44]	1	1	–	1	1	1	1	–
Kim 2008 [33]	1	1	–	1	2	1	1	–
Faro 2005 [28]	1	1	–	1	2	1	1	–
Kishan 2007 [35]	1	1	–	1	2	1	1	–
Lenke 2004 [15]	1	1	–	1	2	1	1	–
Verma 2011 [45]	1	1	–	1	2	1	1	–
Zhou 2011 [49]	1	1	–	1	1	1	1	–
Greggi 2010 [31]	1	1	–	1	2	1	1	–
Lonner 2009 [37]	1	1	–	1	2	1	1	–
Newton 2013 [40]	1	1	–	1	1	1	1	–
Suk 2008 [43]	1	1	–	1	1	1	1	–
Graham 2000 [30]	1	1	–	1	1	1	1	–
Miljenko 2006 [29]	1	1	–	1	2	1	1	–
Lenke 1999 [36]	1	1	–	1	1	1	1	–
Wong 2004 [48]	1	1	–	1	2	1	1	1
Muschik 2006 [39]	1	1	–	1	1	1	1	–
Lonner 2006 [38]	1	1	–	1	2	1	1	–
Hee 2007 [32]	1	1	–	1	2	1	1	–
Wang 2008 [47]	1	1	–	1	2	1	1	–
Dong 2015 [27]	1	1	–	1	2	1	1	–
Zhan 2010 [50]	1	1	–	1	2	1	1	–

*AIS* adolescent idiopathic scoliosis

### Change of absolute FVC

**Fig. 2:**
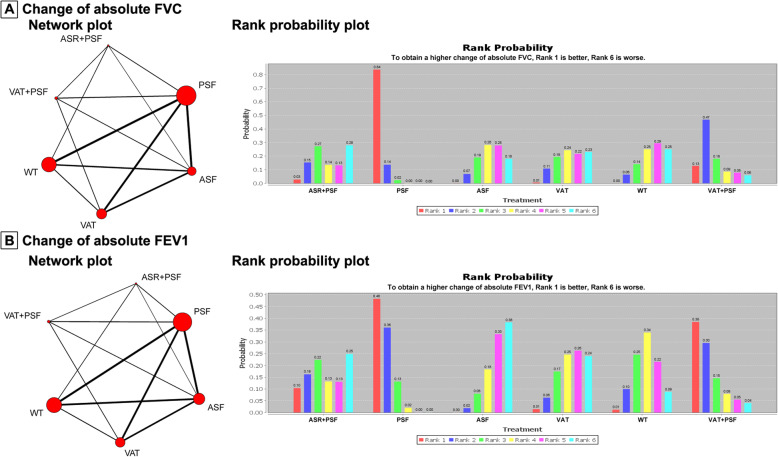
Network and rank probability plots for change of absolute FVC and FEV1. For the network plots: the size of the node corresponds to the total sample size of treatments, directly comparable treatments are linked with a line, and the thickness of which represents the number of trials that were compared. FVC: forced vital capacity; FEV1: forced expiratory volume in 1 s; ASR + PSF: combined anterior release and posterior fusion with instrumentation; VAT+PSF: combined video assisted anterior release and posterior fusion with instrumentation without thoracoplasty; PSF: posterior fusion with instrumentation without thoracoplasty; ASF: anterior fusion with instrumentation and thoracotomy without thoracoplasty; VAT: video assisted anterior fusion with instrumentation without thoracoplasty; WT: any scoliosis surgery with additional thoracoplasty or multiple convex rib resections

Eleven trials were included in this Bayesian meta-analysis. The network of comparisons on change of absolute FVC is shown in Fig. 2a. Table 3 provides the effect sizes on change of absolute FVC from Bayesian meta-analysis and pairwise meta-analysis. Ranking plot of cumulative probability for the change of absolute FVC is shown in Fig. 2a. The Bayesian meta-analysis combining direct and indirect evidences indicated that PSF obtained a greater change of absolute FVC than ASF, VAT or WT. Meanwhile, pairwise meta-analysis also demonstrated the same comparison results between PSF and FVC, ASF, VAT or WT. Moreover, pairwise meta-analysis with limited number of trials indicated three findings that are statistically significant: Change in FVC for PSF > Change in FVC for ASR + PSF; change in FVC for VAT+PSF > Change in FVC for VAT; Change in FVC for VAT > Change in FVC for WT. Based on rank probability from Bayesian meta-analysis, for obtaining a better absolute FVC outcome, PSF ranked first (0.84), followed by VAT+PSF (0.13), ASR + PSF (0.03), VAT (0.01), with ASF (0.00) and WT (0.00) being the last. Therefore, PSF had the highest possibility to obtain a higher change of absolute FVC compared with other interventions.

**Table 3:**
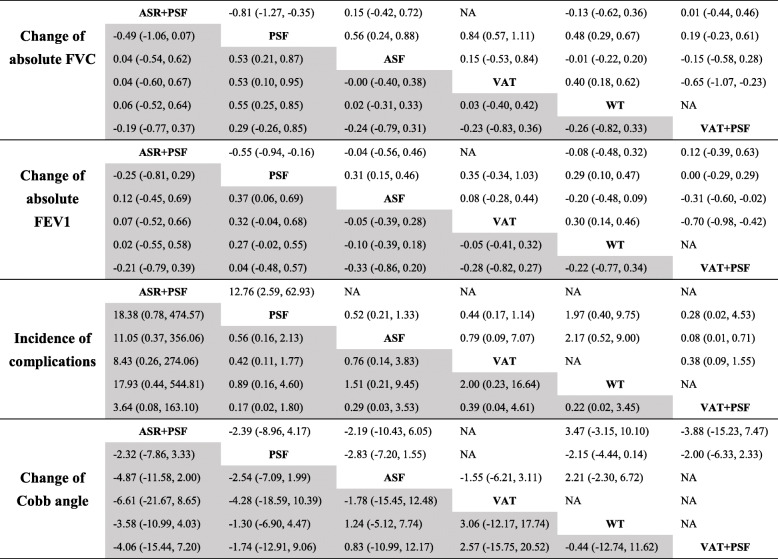
Statistical analysis results from Bayesian meta-analysis (gray background) and pairwise meta-analysis (white background)

*AIS* adolescent idiopathic scoliosis, *FVC* forced vital capacity, *FEV1* forced expiratory volume in 1 s, *ASR + PSF* combined anterior release and posterior fusion with instrumentation, *VAT+PSF* combined video assisted anterior release and posterior fusion with instrumentation without thoracoplasty, *PSF* posterior fusion with instrumentation without thoracoplasty, *ASF* anterior fusion with instrumentation and thoracotomy without thoracoplasty, *VAT* video assisted anterior fusion with instrumentation without thoracoplasty, *WT* any scoliosis surgery with additional thoracoplasty or multiple convex rib resections, *NA* not applicable

### Change of absolute FEV1

Twelve trials were included in this Bayesian meta-analysis. The network of comparisons on change of absolute FEV1 is shown in Fig. 2. Table 3 provides the effect sizes on change of absolute FEV1 from Bayesian meta-analysis and pairwise meta-analysis. Ranking plot of cumulative probability for change of absolute FEV1 are presented in Fig. 2b. Both Bayesian meta-analysis and pairwise meta-analysis demonstrated that PSF had a higher change of absolute FEV1 than ASF. In addition, pairwise meta-analysis with limited number of trials claimed the following: Change in FEV1 of PSF > Change in FEV1 of ASR + PSF or WT; Change in FEV1 for VAT+PSF > Change in FEV1 for VAT; and Change in FEV1 for VAT > Change in FEV1 for WT. Based on rank probability, for obtaining a higher change of absolute FEV1, the ranking of all surgical interventions was: PSF (0.48), VAT+PSF (0.38), ASR + PSF (0.10), VAT (0.01), WT (0.01) and ASF (0.00). PSF turned out to have the highest possibility to obtain a higher change of absolute FEV1 compared with other interventions.

### Incidence of complications

**Fig. 3:**
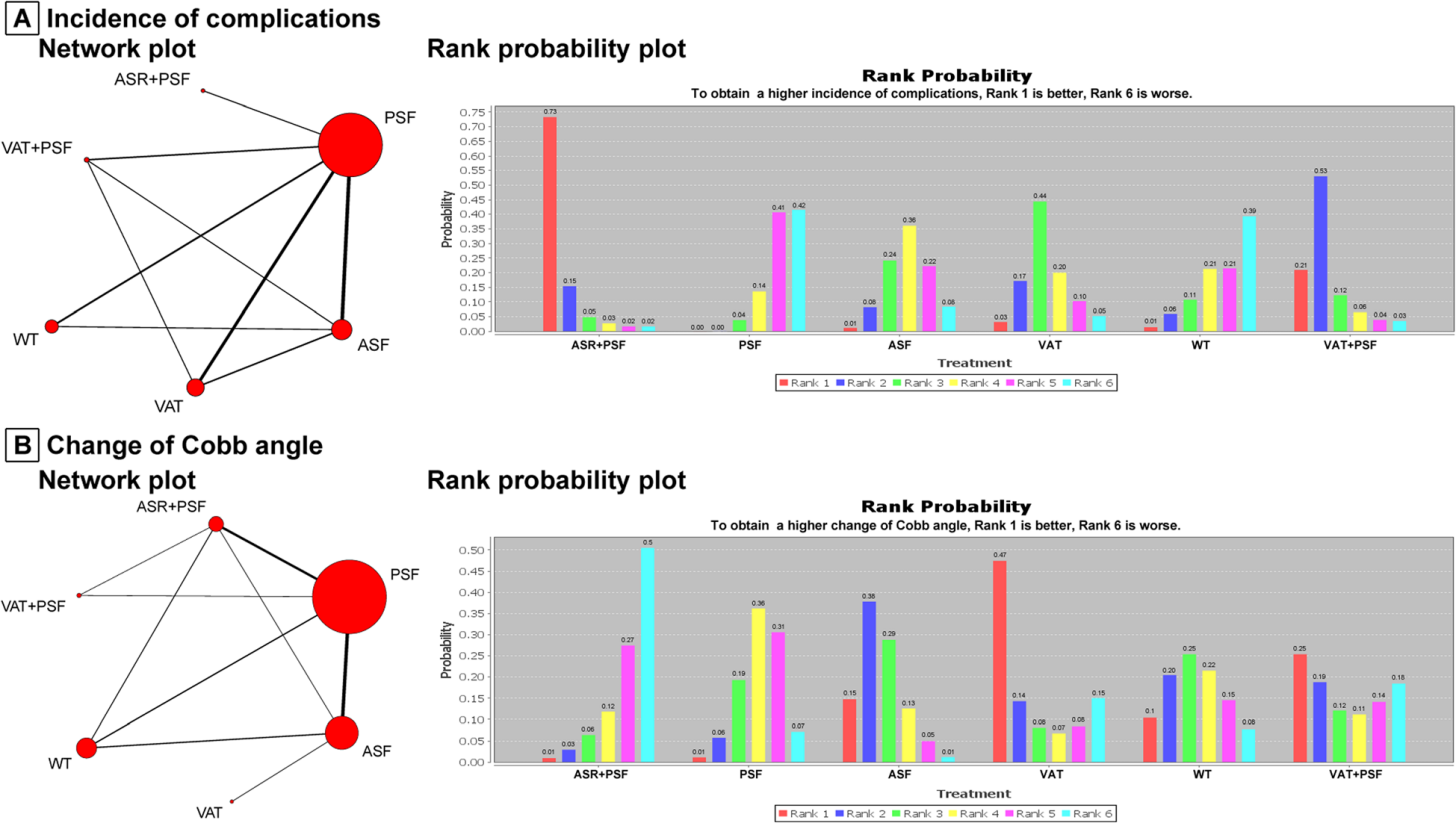
Network and rank probability plots for change of Cobb angle and incidence of complications. For the network plots: the size of the node corresponds to the total sample size of treatments, directly comparable treatments are linked with a line, and the thickness of which represents the number of trials that were compared. ASR + PSF: combined anterior release and posterior fusion with instrumentation; VAT+PSF: combined video assisted anterior release and posterior fusion with instrumentation without thoracoplasty; PSF: posterior fusion with instrumentation without thoracoplasty; ASF: anterior fusion with instrumentation and thoracotomy without thoracoplasty; VAT: video assisted anterior fusion with instrumentation without thoracoplasty; WT: any scoliosis surgery with additional thoracoplasty or multiple convex rib resections

Fourteen trials were included in this Bayesian meta-analysis. The network of comparisons on incidence of complications was shown in Fig. 3a. Table 3 provides the effect sizes on incidence of complications from Bayesian meta-analysis and pairwise meta-analysis. Ranking plot of cumulative probability for incidence of complications is displayed in Fig. 3a. Pairwise meta-analysis with limited number of trials indicated that ASR + PSF resulted in higher incidence of complications than PSF, and there was a higher rate of complications for VAT+PSF compared to ASF. However, Bayesian meta-analysis with 14 trials demonstrated that there was no significant difference among ASR + PSF, VAT+PSF, PSF, ASF, VAT and WT for the incidence of complications. Based on rank probability, for resulting in higher incidence of complications, the ranking of surgical interventions was: ASR + PSF (0.73), VAT+PSF (0.21), VAT (0.03), WT (0.01), ASF (0.01) and PSF (0.00); for obtaining lower incidence of complications, the ranking of surgical interventions was: PSF (0.42), WT (0.39), ASF (0.08), VAT (0.05), VAT+PSF (0.03) and ASR + PSF (0.02). Therefore, ASR + PSF had the highest possibility to obtain higher incidence of complications compared with other interventions; meanwhile, PSF had the highest possibility to obtain lower incidence of complications compared with other interventions.

### Change of cobb angle

Twenty trials were included in this Bayesian meta-analysis. The network of comparisons on change of Cobb angle is shown in Fig. 3b. Table 3 provides the effect sizes on change of Cobb angle from Bayesian meta-analysis and pairwise meta-analysis. Ranking plot presenting the cumulative probability for change of Cobb angle is displayed in Fig. 3b. Both Bayesian meta-analysis and pairwise meta-analysis indicated no statistically significant difference among ASR + PSF, VAT+PSF, PSF, ASF, VAT and WT approaches for the change of Cobb angle. Based on rank probability, to get a higher change of Cobb angle, the ranking of surgical interventions was: VAT (0.47), VAT+PSF (0.25), ASF (0.15), WT (0.10), ASR + PSF (0.01) and PSF (0.01). So VAT had the highest possibility to obtain greater change of Cobb angle compared with other interventions.

### Inconsistency and sensitivity analysis

In general, the results obtained from the pairwise meta-analysis closely matched those from the Bayesian meta-analysis. Table 4 presents the results of inconsistency factors for each outcome. All of the inconsistency factors were close to ‘0’ and the 95% CI covered ‘0’, which indicated that no inconsistency was identified in the Bayesian analysis. Moreover, Node-splitting analysis also demonstrated that there was no inconsistency in Bayesian meta-analysis except for the comparison between PSF and VAT in change of absolute FVC (Table 5). The sensitivity analysis was performed by comparing the random effects standard deviation of different models (consistency and inconsistency model). The random effects standard deviation of consistency model was similar to the inconsistency model, demonstrating statistically robust results for both outcomes (Table 6).

**Table 4:** Inconsistency Factors for each outcome

Outcome	Cycle	Median (95% CrI)
**Change of Cobb angle**	ASR + PSF; PSF; ASF	−0.44 (− 9.14, 5.87)
	ASR + PSF; PSF; ASF; WT	0.73 (−6.35, 11.07)
	ASR + PSF; PSF; VAT+PSF	−0.02 (−9.81, 10.07)
	PSF; ASF; WT	−0.45 (− 10.09, 6.72)
**Incidence of complications**	PSF; ASF; VAT	0.07 (−1.54, 1.92)
	PSF; ASF; VAT; VAT+PSF	−0.06 (− 2.08, 1.63)
	PSF; ASF; WT	−0.04 (− 2.18, 1.66)
	PSF; ASF; VAT+PSF	0.17 (−1.17, 2.62)
**Change of absolute FEV1**	ASR + PSF; PSF; ASF; VAT; VAT+PSF	−0.01 (− 0.45, 0.51)
	ASR + PSF; PSF; VAT; VAT+PSF	0.15 (−0.13, 0.86)
	ASR + PSF; PSF; VAT+PSF	−0.01 (− 0.44, 0.43)
	PSF; ASF; VAT	−0.00 (− 0.53, 0.47)
	PSF; ASF; VAT	−0.00 (− 0.53, 0.47)
	PSF; ASF; VAT; WT	0.01 (− 0.42, 0.55)
	PSF; ASF; VAT; WT	0.01 (−0.42, 0.55)
	ASF; VAT; WT	−0.09 (− 0.68, 0.20)
**Change of absolute FVC**	ASR + PSF; PSF; VAT+PSF	0.05 (− 0.36, 0.77)
	PSF; ASF; VAT; WT	−0.00 (− 0.52, 0.71)
	PSF; ASF; VAT; WT	−0.00 (− 0.52, 0.71)
	PSF; VAT; WT	−0.06 (− 0.76, 0.41)
	PSF; VAT; WT	−0.06 (− 0.76, 0.41)
	ASF; VAT; WT	−0.04 (− 0.76, 0.42)
	ASF; VAT; WT	−0.04 (− 0.76, 0.42)

*ASR + PSF* combined anterior release and posterior fusion with instrumentation, *VAT+PSF* combined video assisted anterior release and posterior fusion with instrumentation without thoracoplasty, *PSF* posterior fusion with instrumentation without thoracoplasty, *ASF* anterior fusion with instrumentation and thoracotomy without thoracoplasty, *VAT* video assisted anterior fusion with instrumentation without thoracoplasty, *W-T* any scoliosis surgery with additional thoracoplasty or multiple convex rib resections, *FEV1* forced expiratory volume in 1 s, *FVC* forced vital capacity

**Table 5:** Node-splitting analysis for inconsistency of Bayesian meta-analysis

Outcome	Comparison	Direct Effect	Indirect Effect	Bayesian Effect	***P***-Value
**Change of Cobb angle**	ASR + PSF vs PSF	2.51 (−3.61, 8.50)	− 0.72 (− 11.35, 9.36)	2.32 (− 3.33, 7.86)	0.54
	ASR + PSF vs ASF	5.00 (− 8.04, 16.68)	3.99 (− 3.65, 11.34)	4.87 (−2.00, 11.58)	0.87
	ASR + PSF vs WT	−1.35 (− 12.88, 9.45)	5.95 (−3.23, 14.94)	3.58 (−4.03, 10.99)	0.27
	ASR + PSF vs VAT+PSF	4.19 (−13.65, 22.04)	4.34 (− 11.59, 19.54)	4.06 (−7.20, 15.44)	1
	PSF vs ASF	2.79 (−1.98, 7.84)	0.68 (−15.13, 16.43)	2.54 (− 1.99, 7.09)	0.79
	PSF vs WT	2.36 (−3.81, 8.75)	2.88 (− 12.15, 17.75)	1.30 (−4.47, 6.90)	0.94
	PSF vs VAT+PSF	2.02 (−11.94, 16.18)	1.52 (−16.87, 20.19)	1.74 (−9.06, 12.91)	0.96
	ASF vs WT	−2.88 (−11.90, 5.60)	1.27 (−8.31, 10.93)	−1.24 (−7.74, 5.12)	0.49
**Incidence of Complications**	PSF vs ASF	0.57 (−1.03, 2.07)	1.14 (−2.80, 5.17)	0.53 (−0.78, 1.76)	0.77
	PSF vs WT	0.26 (−1.73, 2.48)	− 0.38 (−3.99, 3.08)	0.05 (− 1.57, 1.84)	0.72
	ASF vs VAT	0.32 (−2.02, 2.69)	−0.17 (− 2.58, 2.62)	0.26 (− 1.32, 1.96)	0.75
	ASF vs WT	−0.85 (−4.21, 2.55)	− 0.19 (− 2.63, 2.59)	−0.48 (− 2.26, 1.54)	0.73
	ASF vs VAT+PSF	2.17 (−0.74, 5.60)	−1.16 (−5.64, 2.77)	1.08 (− 1.28, 3.41)	0.15
	VAT vs VAT+PSF	1.30 (−1.85, 4.17)	−1.39 (− 5.94, 2.57)	0.84 (− 1.59, 3.08)	0.26
**Change of absolute FEV1**	ASR + PSF vs PSF	0.49 (−0.23, 1.25)	0.14 (−0.48, 0.75)	0.25 (− 0.29, 0.81)	0.4
	ASR + PSF vs ASF	−0.04 (− 0.83, 0.82)	−0.14 (− 0.75, 0.50)	−0.12 (− 0.69, 0.45)	0.84
	ASR + PSF vs WT	0.01 (−0.75, 0.76)	0.02 (−0.60, 0.62)	− 0.02 (− 0.58, 0.55)	0.98
	ASR + PSF vs VAT+PSF	−0.13 (− 0.91, 0.66)	0.56 (− 0.28, 1.40)	0.21 (− 0.39, 0.79)	0.23
	PSF vs ASF	− 0.32 (− 0.72, 0.07)	−0.48 (− 0.91, − 0.03)	−0.37 (− 0.69, − 0.06)	0.56
	PSF vs VAT	− 0.35 (− 0.79, 0.09)	−0.14 (− 0.67, 0.38)	−0.32 (− 0.68, 0.04)	0.51
	PSF vs WT	−0.28 (− 0.64, 0.07)	−0.28 (− 0.79, 0.23)	−0.27 (− 0.55, 0.02)	0.97
	PSF vs VAT+PSF	0.01 (−0.68, 0.70)	−0.56 (− 1.56, 0.47)	−0.04 (− 0.57, 0.48)	0.34
	ASF vs VAT	−0.07 (− 0.41, 0.27)	0.46 (− 0.15, 1.08)	0.05 (− 0.28, 0.39)	0.11
	ASF vs WT	0.18 (−0.16, 0.53)	− 0.04 (− 0.53, 0.44)	0.10 (− 0.18, 0.39)	0.4
	ASF vs VAT+PSF	0.34 (− 0.35, 1.01)	−0.20 (− 1.21, 0.81)	0.33 (− 0.20, 0.86)	0.35
	VAT vs WT	−0.30 (− 0.89, 0.31)	0.20 (− 0.20, 0.61)	0.05 (− 0.32, 0.41)	0.16
	VAT vs VAT+PSF	0.65 (0.00, 1.33)	−0.25 (− 1.28, 0.80)	0.28 (− 0.27, 0.82)	0.15
**Change of absolute FVC**	ASR + PSF vs PSF	0.75 (0.00, 1.50)	0.35 (−0.26, 0.98)	0.49 (−0.07, 1.06)	0.31
	ASR + PSF vs ASF	−0.25 (− 1.03, 0.57)	0.07 (− 0.55, 0.68)	−0.04 (− 0.62, 0.54)	0.44
	ASR + PSF vs WT	0.05 (−0.72, 0.91)	−0.09 (− 0.78, 0.60)	−0.06 (− 0.64, 0.52)	0.72
	ASR + PSF vs VAT+PSF	−0.00 (− 0.77, 0.75)	0.53 (− 0.43, 1.44)	0.19 (− 0.37, 0.77)	0.36
	PSF vs ASF	−0.54 (− 0.98, − 0.14)	−0.58 (− 1.10, − 0.06)	−0.53 (− 0.87, − 0.21)	0.91
	PSF vs VAT	− 0.90 (− 1.26, − 0.48)	−0.17 (− 0.50, 0.16)	−0.53 (− 0.95, − 0.10)	0.02
	PSF vs WT	− 0.47 (− 0.79, − 0.15)	−0.86 (− 1.36, − 0.34)	−0.55 (− 0.85, − 0.25)	0.17
	PSF vs VAT+PSF	− 0.18 (− 0.90, 0.56)	−0.68 (− 1.68, 0.36)	−0.29 (− 0.85, 0.26)	0.4
	ASF vs VAT	−0.15 (− 0.56, 0.26)	0.43 (− 0.19, 1.08)	0.00 (− 0.38, 0.40)	0.1
	ASF vs WT	0.01 (−0.41, 0.46)	− 0.03 (− 0.53, 0.49)	−0.02 (− 0.33, 0.31)	0.9
	ASF vs VAT+PSF	0.22 (−0.56, 0.95)	−0.15 (− 1.18, 0.92)	0.24 (− 0.31, 0.79)	0.54
	VAT vs WT	−0.40 (− 0.96, 0.14)	0.22 (− 0.21, 0.69)	−0.03 (− 0.42, 0.40)	0.07
	VAT vs VAT+PSF	0.57 (−0.15, 1.34)	−0.16 (− 1.25, 0.92)	0.23 (− 0.36, 0.83)	0.23

*ASR + PSF* combined anterior release and posterior fusion with instrumentation, *VAT+PSF* combined video assisted anterior release and posterior fusion with instrumentation without thoracoplasty, *PSF* posterior fusion with instrumentation without thoracoplasty, *ASF* anterior fusion with instrumentation and thoracotomy without thoracoplasty, *VAT* video assisted anterior fusion with instrumentation without thoracoplasty, *W-T* any scoliosis surgery with additional thoracoplasty or multiple convex rib resections, *FEV1* forced expiratory volume in 1 s, *FVC* forced vital capacity

**Table 6:** Random Effects Standard Deviation of Different Models for Sensitivity Analysis

Outcome	Model for Random Effects Standard Deviation	Median (95% CI)
**Change of Cobb angle**	Consistency model	6.28 (4.20, 9.67)
	Inconsistency model	6.40 (4.31, 9.95)
**Incidence of complications**	Consistency model	1.14 (0.02, 2.31)
	Inconsistency model	1.26 (0.38, 2.39)
**Change of absolute FEV1**	Consistency model	0.29 (0.18, 0.50)
	inconsistency model	0.26 (0.11, 0.49)
**Change of absolute FVC**	Consistency model	0.27 (0.13, 0.53)
	Inconsistency model	0.24 (0.07, 0.52)

*FEV1* forced expiratory volume in 1 s, *FVC* forced vital capacity

## Discussion

This Bayesian meta-analysis pooled the data from 28 case-controlled trials, with 1970 participants distributed in ASR + PSF, VAT+PSF, PSF, ASF, VAT or WT groups. To best of our knowledge, this is the first Bayesian meta-analysis combining direct and indirect evidences to provide comprehensive comparisons among multiple surgical interventions for treating AIS that takes 4 criteria into consideration: change of Cobb angle, absolute FVC and absolute FEV1 from pre-operation to post-operation, and incidence of complications. In this analysis, we found that PSF had the highest possibility to obtain a greater change of absolute FVC and FEV1 and a lower incidence of complications compared with other interventions based on rank probability. Moreover, VAT had the highest possibility to obtain greater change of Cobb angle.

Based on the quality assessment evaluated by Newcastle-Ottawa Scale, 9 studies were scored 6 with all others scored at least 7, suggesting that the included studies have moderate or high quality. In the node-splitting analysis, the only inconsistency was found between PSF and VAT when comparing change of absolute FVC. We checked the inclusion and statistical processes to find the reason for the inconsistency and noticed that only one study reported this comparison. However, the results of inconsistency factors demonstrated that there was no inconsistency in this Bayesian meta-analysis. In general, the results from this Bayesian meta-analysis were reliable and robust.

Spinal deformity can profoundly affect pulmonary function by alternation of lung development, which may cause early mortality through respiratory failure [53]. So early interventions are recommended to prevent and correct the development of the spinal deformity. FVC and FEV1 were considered as two common parameters to assess the patients’ pulmonary functions pre-operatively and post-operatively. Several traditional meta-analyses comparing only two surgical interventions for FVC and FEV1 have been published. Chen et al. [2] reported that posterior surgery could achieve similar improvement in percent-predicted FVC compared to combined anterior-posterior surgery. Lee et al. [16] found that posterior spinal fusion with instrumentations resulted in small to moderate increases in FVC and FEV1. It seems that posterior surgery gave better pulmonary function than anterior or combined anterior-posterior surgeries. As shown in this present Bayesian meta-analysis, PSF obtained a greater change of absolute FVC than ASF, VAT or WT, and also had a greater change of absolute FEV1 than ASF, which is in agreement of the previous reports. Moreover, based on rank probability, PSF had the highest possibility to obtain greater change of absolute FVC and FEV1 compared with other five surgical interventions. The results also reproduced a reported trend that the amount of PSF surgery is increasing year by year, while the amount of thoracoplasty is decreasing gradually [54].

Incidence of complications is important to evaluate the safety of different surgical interventions. Chen et al. [2] previously reported that posterior-only surgery achieved lower complication rate compared to combined anterior-posterior surgery. Lonner et al. [54] performed a retrospective review of the prospective AIS registry and demonstrated that as the amount of PSF surgery increases, the incidence of complications gradually decreases from 1995 to 2013. Those studies comparing limited kinds of surgical interventions indicated that posterior surgery might achieve lower complication rate. However, our Bayesian meta-analysis including 14 trials revealed that there was no statistically significant difference among ASR + PSF, VAT+PSF, PSF, ASF, VAT and WT in incidence of complications. But we still found that PSF had the highest possibility to obtain lower incidence of complications compared with other five surgical interventions based on rank probability, which agreed to the previous studies. This may be attributed to the low implant-related complications of posterior pedicle-screw and enhanced surgeon experiences [54, 55].

Cobb angle measurement is a traditional method to assess the spine deformity of AIS, which is carried out in the coronal plane using a standard postero-anterior radiograph [5]. Previous studies focused on discussing the coronal plane correction between anterior and posterior surgery. Luo et al. [56] reported that the posterior approach can obtain a larger change of Cobb angle from pre-operation to final follow-up. Franic et al. [17] found that both anterior and posterior surgeries provided a similar degree of reduction of frontal Cobb angle, and long-term effects of surgical correction on the sagittal Cobb angle seemed to be more stable in posterior group. However, in this study, both Bayesian meta-analysis and pairwise meta-analysis indicated no statistically significant difference among ASR + PSF, VAT+PSF, PSF, ASF, VAT and WT approaches for the change of Cobb angle. Furthermore, the rank probability of outcomes was used to distinguish the subtle differences of change in Cobb angle among the six interventions, which revealed that VAT had the highest possibility to obtain greater change of Cobb angle. In addition, VAT also resulted in less invasive, fewer levels fused and better satisfaction [57]. However, it had a long learning curve and specific indications. Therefore, with appropriate training and careful patient selection, VAT might be a more effective surgical intervention compared to traditional surgical interventions.

Refering to the classification system, Lenke classification system is the most common classification system for AIS, but it can not definitely decide the surgery strategies. Among the RCTs which we included, most of them didn’t discuss the classification of AIS, they just simply defined severe AIS as the Cobb’s angle for main curve≥90°. So, we only focus on the choice of the surgery approaches for AIS in this analysis. The selection of the upper instrumented vertebra (UIV), lower instrumented vertebra (LIV) and the instrumented segment was not taken into our consideration. For the surgery approaches, we suggest that PSF still is the primary choice for AIS because of the minimal influence on pulmonary function and low complication rate. Moreover, the deformity correction rate of PSF is comparable to the other surgery approaches. However, anterior approach combined with posterior approach might be necessary to the patients with severe AIS for maximum correction of deformity. Compared to ASF approach, VAT approach was more more minimally invasive, and has advantages in deformity correction because of less damage to the tissue and less blood loss. However, ASF approach is also a choice for AIS because the VAT approach has a long learning curve for surgeons. The VAT+PSF approach could deal with the severe AIS, which is the tendency for surgery approaches nowadays. And the WT approach should be avoided as as much as possible because of the adverse effects on pulmonary function.

This Bayesian meta-analysis has several limitations. Firstly, this study only included case-controlled studies, because randomized controlled trials are challenging to perform in pediatric population [58], and the case-controlled studies may have reduced the quality of the evidences; Secondly, because of the limited number of included studies, we did not distinguish different kind of complications, such as infection, neurologic deficit and instrumentation failure. Instead, all of the complications reported in the included studies were recorded as incidence of complications for statistical analysis; Finally, though Lenke classification is a useful scale to guide the treatment of AIS, this analysis did not distinguish different Lenke types of AIS but rather involve all the cases into one integral analysis, due to the limited number of included studies. This might be potential bias to the outcomes. However, this Bayesian meta-analysis still provided useful information on effectiveness and safety of surgical interventions for treating AIS to the surgeons.

## Conclusion

In summary, this Bayesian meta-analysis demonstrated that PSF had the highest probability to achieve better post-surgical pulmonary function and lower complication rate. Moreover, VAT was believed to have the highest probability to obtain better Cobb angle correction. These results support the current statistics, that more surgeries adopted PSF and less surgeries adopted open anterior approach surgery and thoracoplasty. This analysis also gives a practical recommendation of PSF as a primary surgical treatment for AIS. More research work is required to address the effectiveness and safety of VAT for treating AIS more convincingly.

## Supplementary information

**Additional file 1: Table S1.** The Newcastle-Ottawa Scale for assessing the quality of case-controlled studies in meta-analyses.

## Data Availability

Not applicable.

## Abbreviations

AIS: Adolescent idiopathic scoliosis
3D: Three-dimensional
ASR + PSF: Combined anterior release and posterior fusion with instrumentation
VAT+PSF: Combined video assisted anterior release and posterior fusion with instrumentation without thoracoplasty
PSF: Posterior fusion with instrumentation without thoracoplasty
AS: Anterior fusion with instrumentation and thoracotomy without thoracoplasty
VAT: Video assisted anterior fusion with instrumentation without thoracoplasty
WT: Any scoliosis surgery with additional thoracoplasty or multiple convex rib resections
FVC: Absolute forced vital capacity
FEV1: Absolute forced expiratory volume in one second
PRISMA: Preferred Reporting Items for Systematic Reviews and Meta-Analyses
OR: Odds ratio
MDs: Mean differences
CI: Confidence interval
PSRF: Potential scale reduction factor
